# Robot-Assisted Versus Laparoscopic Distal Pancreatectomy in Patients with Resectable Pancreatic Cancer: An International, Retrospective, Cohort Study

**DOI:** 10.1245/s10434-022-13054-2

**Published:** 2023-02-17

**Authors:** Jeffrey W. Chen, Tess M. E. van Ramshorst, Sanne Lof, Bilal Al-Sarireh, Bergthor Bjornsson, Ugo Boggi, Fernando Burdio, Giovanni Butturini, Riccardo Casadei, Andrea Coratti, Mathieu D’Hondt, Safi Dokmak, Bjørn Edwin, Alessandro Esposito, Jean M. Fabre, Giovanni Ferrari, Fadhel S. Ftériche, Giuseppe K. Fusai, Bas Groot Koerkamp, Thilo Hackert, Asif Jah, Jin-Young Jang, Emanuele F. Kauffmann, Tobias Keck, Alberto Manzoni, Marco V. Marino, Quintus Molenaar, Elizabeth Pando, Patrick Pessaux, Andrea Pietrabissa, Zahir Soonawalla, Robert P. Sutcliffe, Lea Timmermann, Steven White, Vincent S. Yip, Alessandro Zerbi, Mohammad Abu Hilal, Marc G. Besselink, For the European Consortium on Minimally Invasive Pancreatic Surgery (E-MIPS), Beatrice Aussilhou, Sivesh K. Kamarajah, Stijn van Laarhoven, Thomas Malinka, Ravi Marudanayagam, Claudio Ricci, Patricia Sánchez-Velázquez

**Affiliations:** 1 Amsterdam UMC, Department of Surgery, Location University of Amsterdam, Amsterdam, The Netherlands; 2 Cancer Center Amsterdam, Amsterdam, The Netherlands; 3 Department of General Surgery, Istituto Ospedaliero Fondazione Poliambulanza, Brescia, Italy; 4 Department of Surgery, Morriston Hospital, Swansea, UK; 5 Department of Surgery and Department of Biomedical and Clinical Sciences, Linköping University, Linköping, Sweden; 6 Department of Surgery, University Hospital of Pisa, Pisa, Italy; 7 Department of Surgery, University Hospital del Mar, Barcelona, Spain; 8 Department of Surgery, Pederzoli Hospital, Peschiera, Italy; 9 Department of Surgery, Sant’Orsola Malphigi Hospital, Bologna, Italy; 10 Division of Oncological and Robotic General Surgery, Careggi University Hospital, Florence, Italy; 11 Department of Digestive and Hepatobiliary/Pancreatic Surgery, Groeninge Hospital, Kortrijk, Belgium; 12 Department of HPB Surgery and Liver Transplantation, Beaujon Hospital, Clichy, France; 13 The Intervention Center, Department of Surgery, Oslo University Hospital and Institute of Clinical Medicine, University of Oslo, Oslo, Norway; 14 Department of General and Pancreatic Surgery, Pancreas Institute, Verona University Hospital, Verona, Italy; 15 Department of Surgery, Saint-Éloi Hospital, Montpellier, France; 16 Department of Surgery, Niguarda Hospital, Milan, Italy; 17 HPB & Liver Transplant Unit, Royal Free London, London, UK; 18 Department of Surgery, Erasmus MC Cancer Institute, Rotterdam, The Netherlands; 19 Department of Surgery, Heidelberg University Hospital, Heidelberg, Germany; 20 Department of HPB Surgery and Transplantation, Cambridge University Hospitals NHS Foundation Trust, Cambridge, UK; 21 Department of Surgery, Seoul National University Hospital, Seoul, South Korea; 22 Department of Surgery, University Medical Center Schleswig-Holstein, Campus Lübeck, Lübeck, Germany; 23 Department of Emergency and General Surgery, Azienda Ospedaliera Ospedali Riuniti Villa Sofia-Cervello, Palermo, Italy; 24 Department of Surgery, UMC Utrecht Cancer Center, University Medical Center Utrecht, Utrecht, The Netherlands; 25 Department of Surgery, Vall d’Hebron University Hospital, Barcelona, Spain; 26 Department of Hepato-Biliary and Pancreatic Surgery, Nouvel Hôpital Civil, Institut Hospitalo-Universitaire de Strasbourg, Strasbourg, France; 27 Department of Surgery, Fondazione IRCCS Policlinico San Matteo, Pavia, Italy; 28 Department of Surgery, Oxford University Hospital, Oxford, UK; 29 Department of Hepato-Pancreato-Biliary and Liver Transplant Surgery, Queen Elizabeth University Hospitals Birmingham, Birmingham, UK; 30 Department of Surgery, Charité, Berlin, Germany; 31 Department of Surgery, The Freeman Hospital, Newcastle Upon Tyne, Newcastle, UK; 32 Department of HPB Surgery, The Royal London Hospital, Bartshealth NHS Trust, London, UK; 33 Department of Surgery, Humanitas University and IRCCS Humanitas Research Hospital, Rozzano, Milan, Italy

## Abstract

**Background:**

Robot-assisted distal pancreatectomy (RDP) is increasingly used as an alternative to laparoscopic distal pancreatectomy (LDP) in patients with resectable pancreatic cancer but comparative multicenter studies confirming the safety and efficacy of RDP are lacking.

**Methods:**

An international, multicenter, retrospective, cohort study, including consecutive patients undergoing RDP and LDP for resectable pancreatic cancer in 33 experienced centers from 11 countries (2010–2019). The primary outcome was R0-resection. Secondary outcomes included lymph node yield, major complications, conversion rate, and overall survival.

**Results:**

In total, 542 patients after minimally invasive distal pancreatectomy were included: 103 RDP (19%) and 439 LDP (81%). The R0-resection rate was comparable (75.7% RDP vs. 69.3% LDP, *p* = 0.404). RDP was associated with longer operative time (290 vs. 240 min, *p* < 0.001), more vascular resections (7.6% vs. 2.7%, *p* = 0.030), lower conversion rate (4.9% vs. 17.3%, *p* = 0.001), more major complications (26.2% vs. 16.3%, *p* = 0.019), improved lymph node yield (18 vs. 16, *p* = 0.021), and longer hospital stay (10 vs. 8 days, *p* = 0.001). The 90-day mortality (1.9% vs. 0.7%, *p* = 0.268) and overall survival (median 28 vs. 31 months, *p* = 0.599) did not differ significantly between RDP and LDP, respectively.

**Conclusions:**

In selected patients with resectable pancreatic cancer, RDP and LDP provide a comparable R0-resection rate and overall survival in experienced centers. Although the lymph node yield and conversion rate appeared favorable after RDP, LDP was associated with shorter operating time, less major complications, and shorter hospital stay. The specific benefits associated with each approach should be confirmed by multicenter, randomized trials.

Minimally invasive distal pancreatectomy (MIDP) has become the preferred approach for most resectable lesions in the pancreatic body and tail.^1^ Two randomized trials and numerous retrospective studies have shown that MIDP, consisting of both laparoscopic distal pancreatectomy (LDP) and robot-assisted distal pancreatectomy (RDP), is associated with faster functional recovery compared with open distal pancreatectomy (ODP).^2–4^ Although MIDP is increasingly being used in patients with resectable pancreatic cancer,^5,6^ randomized, controlled trials confirming its safety and efficacy in this patient category are still lacking.

While MIDP is mainly performed through laparoscopy, the robot-assisted approach is gaining popularity.^7^ In general, for all indications, retrospective studies have suggested that RDP is associated with improved rates of spleen-preservation and conversion, and shorter hospital stay, compared with LDP.^7–10^ However, studies specifically in patients with pancreatic cancer are scarce and only consist of single-center or small, cohort studies.^11,12^

To date, an international comparison of RDP and LDP in a large cohort of patients with resectable pancreatic cancer in experienced centers is lacking. As the use of a robotic approach in distal pancreatectomy continues to increase, it is important to investigate its safety and efficacy in patients with pancreatic cancer. Therefore, the purpose of this study is to compare the surgical and oncological outcome of RDP versus LDP in patients with resectable pancreatic cancer in a large, international, multicenter cohort.

## Methods

### Study Population and Design

This retrospective study was performed among centers participating in the European Consortium on Minimal Invasive Pancreatic Surgery (E-MIPS) and one non-European center. Only centers who had performed at least 50 MIDP procedures for all indications were included. Consecutive patients undergoing MIDP for pancreatic ductal adenocarcinoma (PDAC) between January 1, 2010 and December 31, 2019 were screened for eligibility. Patients were excluded if they had a previous pancreatic resection or were considered as borderline or locally advanced pancreatic cancer at diagnosis according to the NCCN guidelines.^13^ Patients were categorized according to the surgical technique applied: RDP and LDP. Patients undergoing conversion were included according to the initial surgical approach. Primary outcome was the R0-resection rate. Secondary outcomes included lymph node yield, major complication rate, conversion rate, and overall survival.

At each participating center, a local coordinator was responsible for the communication with the central study coordinators (JC, SL). Participating centers provided anonymized data on a password secured database. The central study coordinators combined the data.

This study was performed according to the principles of the Declaration of Helsinki (64th Fortaleza Brazil, October 2013) and in accordance with the Medical Research Involving Human Subjects Act (WMO) and STROBE guidelines on reporting on observational studies.^14^ Due to the retrospective design, the ethical board from Amsterdam UMC waived the need for informed consent.

### Definitions

Pancreatic cancer was defined according to the WHO classification of pancreatic tumors as pancreatic ductal adenocarcinoma.^15^ Conversion was defined as any attempted minimally invasive resection requiring conversion to laparotomy for other reasons than trocar placement or specimen extraction.^16^ Conversions were classified as elective conversions if there were unexpected findings, such as progression of tumor into surrounding structures or difficulty achieving tumor exposure or dissection. Conversions were classified as emergency conversion if unexpected events occurred, for instance bleeding.^10^ Operation time was calculated from robotic docking until completion of the surgical procedure. Postoperative complications were classified according to the Clavien-Dindo classification.^17^ Major complications were defined as Clavien-Dindo grades ≥3a. The definitions of pancreatic surgery specific complications of the International Study Group of Pancreatic Surgery were used to define postoperative pancreatic fistula, delayed gastric emptying, and post-pancreatectomy hemorrhage.^18–20^ Only complications graded as B and C were noted. Data on surgical site infections or radiological interventions were not collected. Postoperative outcomes were recorded up to 90 days postoperatively. Resection margins, including transection and posterior margins, were by all centers similarly categorized into: R0 (distance margin to tumor ≥1 mm), R1 (distance margin to tumor <1 mm), and R2 (macroscopically positive margin) according to the Royal College of Pathologists definition.^21^ The tumors were classified according to the American Joint Committee on Cancer (AJCC) 8th edition staging system.^22^

### Statistical Analysis

Data were analyzed by using IBM SPSS Statistics for Windows version 26.0 (IBM Corp., Orchard Road Armonk, NY). Analyses were performed according to the intention-to-treat principle. Normally distributed continuous data were presented as mean with standard deviations (SD) and were compared by using the two-tailed Student *t*-test. Nonnormally distributed continuous data were presented as median with interquartile range (IQR) and were compared using the Mann-Whitney *U* test or the Kruskal–Wallis test, as appropriate. Categorical data were presented as frequencies with percentages and were compared by using the chi-square or Fisher’s exact test, as appropriate. The overall survival and disease-free interval were calculated by using Kaplan-Meier estimates and reported until 36 months of follow-up. Overall survival was defined from the date of surgery until the date of death or loss of follow-up, and all patients who were alive at the last follow-up date were censored. Disease-free interval was defined from the date of surgery until the first recurrence or death. The log-rank (Mantel-Cox) test was used to compare survival probabilities. *P* < 0.05 was considered statistically significant. Additionally, multivariable logistic regression analyses were performed for the two main outcomes of the study: R0 resection and major complications to examine whether the surgical approach or other variables were significantly associated with both outcomes. Variables with *p* < 0.20 in univariable analysis or clinical relevance based on literature were considered for multivariable analysis. Multivariable logistic regression analysis was performed by using binary logistic regression with backward selection with a *p* < 0.10, presented as odds ratios (OR) with corresponding 95% confidence intervals (CI). *P* < 0.05 was considered statistically significant.

## Results

Overall, 542 patients after MIDP for resectable pancreatic cancer were included from 33 centers in 11 countries. Of the 542 patients, 103 patients (19%) underwent RDP and 439 patients (81%) LDP, without any differences in baseline characteristics between both groups (Table 1).

**Table 1:** Baseline characteristics of patients undergoing RDP and LDP for resectable pancreatic cancer

	RDP (*n* = 103)	LDP (*n* = 439)	*p*
Age, yr, median (IQR)	70 (62–74)	70 (63–76)	0.566
Age ≥ 65, *n* (%)	72 (69.9)	305 (69.5)	0.932
Female sex, *n* (%)	45 (43.7)	231 (52.6)	0.103
BMI, kg/m^2^, median (IQR)	24.4 (22.0–27.1)	24.3 (22.3–27.1)	0.649
BMI ≥30, *n* (%)	9 (9.3)	53 (13.3)	0.288
ASA III–IV, *n* (%)	41 (40.6)	147 (34.0)	0.214
Prior abdominal surgery, *n* (%)	33 (32.0)	182 (41.7)	0.070
Preoperative tumor size, mm, median (IQR)	27.0 (20.0–32.0)	25.0 (20.0–35.0)	0.955
Size≥50 mm, *n* (%)	6 (7.1)	35 (9.8)	0.454
Neoadjuvant chemotherapy, *n* (%)	10 (10.5)	34 (8.5)	0.528
Operation period 2010–2014/2015–2019, *n* (%)	20 (19.4)/83 (80.6)	127 (28.9)/312 (71.1)	0.051

Percentages may not add up due to rounding and missing data

*RDP* robot-assisted distal pancreatectomy, *LDP* laparoscopic distal pancreatectomy, *IQR* interquartile range, *BMI* body mass index, *ASA* American Society of Anesthesiologists

### Intraoperative Outcomes

Intraoperative variables are presented in Table 2. RDP was associated with a longer operative time (290 vs. 240 minutes, *p* < 0.001) and more vascular resections (7.6% vs. 2.7%, *p* = 0.030). The rate of conversion to open surgery was significantly lower in the RDP group (4.9% vs. 17.3%, *p* = 0.001). No emergency conversions occurred during RDP compared with LDP (0% vs. 5.3%, *p* = 0.004). Both emergency conversions and elective conversions required longer operating time compared with procedures without conversion (295 and 280 vs. 234 minutes, *p* < 0.001).

**Table 2:** Intraoperative variables of patients undergoing RDP and LDP for resectable pancreatic cancer

	RDP (*n* = 103)	LDP (*n* = 439)	*p*
Operative time, min, median (IQR)	290 (210–338)	240 (170–300)	< 0.001
Blood loss, ml, median (IQR)	200 (100–300)	173 (100–300)	0.378
Blood transfusion intraoperative, *n* (%)	5 (5.3)	25 (6.4)	0.681
Vascular resection, *n* (%)	6 (7.6)	11 (2.7)	0.030
PV/SMV, *n* (%)	0 (0)	7 (1.7)	
Other (e.g., renal vein), n (%)	6 (7.6)	4 (1.0)	
Multivisceral resection, *n* (%)	7 (8.9)	52 (12.6)	0.350
Splenectomy, *n* (%)	94 (92.2)	405 (92.3)	0.973
Conversion, *n* (%)	5 (4.9)	76 (17.3)	0.001
Elective conversion, *n* (%)	4 (3.9)	47 (10.9)	
Emergency conversion, *n* (%)	0 (0)	23 (5.3)	0.004
Intraoperative drain placement, *n* (%)	96 (98.0)	435 (99.1)	0.336

Percentages may not add up due to rounding and missing data

*RDP* robot-assisted distal pancreatectomy, *LDP* laparoscopic distal pancreatectomy, *IQR* interquartile range, *PV* portal vein, *SMV* superior mesenteric vein

### Histopathological Outcomes

Histopathological variables are shown in Table 3. The R0-resection rate did not differ between RDP and LDP (75.7% vs. 69.3%, *p* = 0.404). The median lymph node yield was higher in RDP compared with LDP (18 vs. 16, *p* = 0.021), whereas no difference was observed in rate of positive lymph nodes between both groups (58.2% vs. 59.6%, *p* = 0.799).

**Table 3:** Postoperative pathological and oncological outcome patients undergoing RDP and LDP for resectable pancreatic cancer

		RDP (n = 103)	LDP (n = 439)	*p*
Size of lesion, mm, median (IQR)		30 (21.8–40.0)	30 (21.0–40.0)	0.849
Lymph node retrieval, n, median (IQR)		18 (13–28)	16 (10–25)	0.021
Patients with positive lymph nodes, n (%)		252 (58.2)	59 (59.6)	0.799
Positive lymph nodes, n, median (range)		1 (0-16)	1 (0-26)	0.562
Tumor differentiation, n (%)				0.602
Well, n, (%)		13 (13.1)	63 (17.2)	
Moderate, n (%)		59 (59.6)	203 (55.5)	
Poor, n (%)		27 (27.3)	97 (26.5)	
Undifferentiated, n (%)		0 (0)	3 (0.8)	
Tumor stage (8th AJCC), n (%)				0.442
T	1, n (%)	18 (17.8)	105 (24.1)	
	2, n (%)	60 (59.4)	224 (51.5)	
	3, n (%)	23 (22.7)	105 (24.1)	
	4, n (%)	0(0.0)	1 (0.23)	
Lymph node stage (8th AJCC), n (%)				0.968
N	0, n (%)	40 (40.4)	181 (41.8)	
	1, n (%)	43 (43.4)	184 (42.5)	
	2, n (%)	16 (16.2)	68 (15.7)	
Metastatic stage (8th AJCC), n (%)				0.255
M	0, n (%)	82 (94.3)	308 (96.9)	
	1, n (%)	5 (5.7)	10 (3.1)	
R0 resection, n (%)		78 (75.7)	298 (69.3)	0.404
Adjuvant chemotherapy, n (%)		62 (77.5)	254 (72.6)	0.368
Recurrence, n (%)		34 (43.0)	179 (51.0)	0.201
Length of follow-up, median (IQR)		12 (6–21)	18 (10–30)	< 0.001
Overall survival, mo, median		28	31	0.602
1-yr, overall survival, %		79.4	81.5	–
3-yr, overall survival, %		43.7	46.6	–
Disease-free interval, mo, median		21	25	0.366
1-yr, disease-free interval, %		67.1	70.7	–
3-yr, disease-free interval, %		30.8	35.7	–

Percentages may not add up due to rounding and missing data

*RDP* robot-assisted distal pancreatectomy, *LDP* laparoscopic distal pancreatectomy, *IQR* interquartile range, *AJCC* American Joint Committee on Cancer, *mo* months

### Postoperative Outcome

Postoperative outcomes are presented in Table 4. Major complications occurred more frequently after RDP (26.2% vs. 16.3%, *p* = 0.019), whereas the rate of postoperative pancreatic fistula grade B/C (20.4% vs. 19.4%, *p* = 0.821), post-pancreatectomy hemorrhage grade B/C (2.9% vs. 3.0%, *p* = 0.953), and delayed gastric emptying (4.0% vs. 1.7%, *p =* 0.144) did not differ significantly between RDP and LDP, respectively. The median length of hospital stay was longer after RDP (10 vs. 8 days, *p* = 0.001). No differences were found in readmission and reoperation rates between both groups. The 30-day mortality (1.9% vs. 0.7%, *p* = 0.241) and 90-day mortality (1.9% vs. 0.7%, *p* = 0.268) did not differ between RDP and LDP, respectively.

**Table 4:** Postoperative surgical outcome after RDP and LDP for resectable pancreatic cancer

	RDP (*n* = 103)	LDP (*n* = 439)	*p*
Length of stay, days, median (IQR)	10 (7–15)	8 (6–12)	0.001
Drain removal, days, median (IQR)	7 (5–14)	6 (4–11)	0.182
Clavien-Dindo grade ≥3a, *n* (%)	27 (26.2)	71 (16.3)	0.019
Blood transfusion postoperative, *n* (%)	8 (8.4)	45 (10.7)	0.502
POPF grade B/C, *n* (%)	21 (20.4)	85 (19.4)	0.821
PPH grade grade B/C, *n* (%)	3 (2.9)	13 (3.0)	0.953
DGE grade B/C, *n* (%)	4 (4.0)	7 (1.7)	0.144
Reoperation, *n* (%)	8 (7.8)	22 (5.0)	0.271
Readmission, *n* (%)	10 (10.0)	59 (13.7)	0.327
30-day mortality, *n* (%)	2 (1.9)	3 (0.7)	0.241
90-day mortality, *n* (%)	2 (1.9)	3 (0.7)	0.268

Percentages may not add up due to rounding and missing data

*RDP* robot-assisted distal pancreatectomy, *LDP* laparoscopic distal pancreatectomy, *IQR* interquartile range, *POPF* postoperative pancreatic fistula, *PPH* post-pancreatectomy hemorrhage, *DGE* delayed gastric emptying

**Fig. 1:**
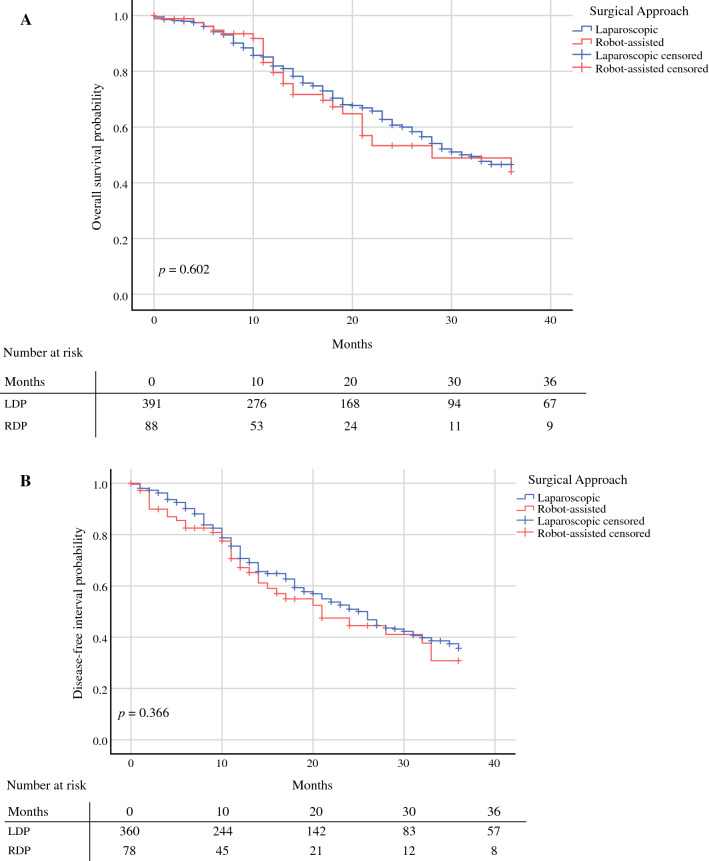
**A** Kaplan-Meier curve of overall survival in patients with resectable pancreatic cancer after robot-assisted distal pancreatectomy (RDP) and laparoscopic distal pancreatectomy (LDP). **B** Kaplan-Meier curve of disease-free interval of patients after robot-assisted distal pancreatectomy (RDP) and laparoscopic distal pancreatectomy (LDP)

The median follow-up time was 12 months (interquartile range [IQR] 6–12) for RDP and 18 months (IQR 10-30) for LDP. No differences were observed between both groups in overall survival (median RDP 28 vs. LDP 31 months, *p* = 0.602), as shown in Fig. 1A, and the disease-free interval (median RDP 21 vs. LDP 25 months, *p* = 0.366), as shown in Fig. 1B.

### Multivariable Regression Analyses

In the multivariable regression analysis of R0 resection, age ≥65 years (OR 1.86, 95% CI 1.11–3.13, *p* = 0.019), ASA classification of III–IV (OR 1.68, 95% CI 1.07–2.64, *p* = 0.024), intraoperative blood transfusion (OR 2.51, 95% CI 1.09–5.78, *p* = 0.031), and elective conversion (OR 2.39, 95% CI 1.25–4.58, *p* = 0.008) were associated risk factors for a R1 resection (Table 5).

**Table 5:** Uni- and multivariable logistic regression analysis of R0 resection

Variable	Univariable analysis		Multivariable analysis	
	OR (95% CI)	*p*	OR (95% CI)	*p*
Age >65 yr vs. age <65 yr	1.84 (1.20–2.84)	0.006	1.86 (1.11–3.13)	0.019
Approach robotic vs. laparoscopic	0.72 (0.44–1.17)	0.185		*Removed on step 7*
ASA III-IV vs. ASA I-II	1.99 (1.35–2.92)	<0.001	1.68 (1.07–2.64)	0.024
BMI >30 vs. BMI <30	1.18 (0.67–2.09)	0.573		*Removed on step 2*
Multivisceral resection yes vs. no	1.35 (0.76–2.40)	0.302		*Removed on step 3*
Vascular resection yes vs. no	1.29 (0.47–3.55)	0.627		*Removed on step 4*
Neoadjuvant therapy yes vs. no	1.73 (0.91–3.28)	0.096		*Removed on step 6*
Blood transfusion intraoperative yes vs. no	3.17 (1.50–6.69)	0.002	2.51 (1.09–5.78)	0.031
T stage 3-4 vs. T stage 1-2	1.55 (1.02–2.36)	0.042		*Removed on step 5*
Conversion, categorized				
No conversion	Ref.			
Elective conversion	2.64 (1.47–4.75)	0.001	2.39 (1.25–4.58)	0.008
Emergency conversion	1.20 (0.48–2.99)	0.695	1.10 (0.40–3.04)	0.853

*BMI* body mass index, *OR* odds ratio, *ASA* American Society of Anesthesiologists

Multivariable logistic regression analysis of potential variables associated with major complications revealed that only an ASA classification of III–IV was significantly associated with an increased risk of major complications (OR 1.81, 95% CI 1.09–3.00, *p* = 0.021) as shown in Table 6. RDP was not an associated risk factor when adjusted for other variables (OR 1.41, 95% CI 0.75–2.65, *p* = 0.29).

**Table 6:** Uni- and multivariable logistic regression analysis of major complications

Variable	Univariable analysis		Multivariable analysis	
	OR (95% CI)	*p*	OR (95% CI)	*p*
Age >65 yr vs. age <65 yr	1.06 (0.66–1.71)	0.798		*Removed on step 3*
Approach robotic vs. laparoscopic	1.82 (1.10–3.03)	0.021		*Removed on step 5*
ASA III-IV vs. ASA I-II	1.43 (0.92–2.25)	0.115	1.81 (1.09–3.00)	0.021
BMI >30 vs. BMI <30	1.54 (0.82–2.91)	0.181		*Removed on step 6*
Multivisceral resection yes vs. no	1.43 (0.73–2.77)	0.297		*Removed on step 7*
Vascular resection yes vs. no	1.46 (0.46–4.59)	0.518		*Removed on step 2*
Neoadjuvant therapy yes vs. no	0.68 (0.28–1.67)	0.405		*Removed on step 9*
Blood transfusion intraoperative yes vs. no	1.99 (0.88–4.51)	0.098		*Removed on step 4*
T stage 3-4 vs. T stage 1-2	1.43 (0.88–2.33)	0.153		*Removed on step 10*
Conversion, categorized				*Removed on step 8*
No conversion	Ref.			
Elective conversion	1.76 (0.91–3.40)	0.094		
Emergency conversion	0.70 (0.20–2.40)	0.566		

*BMI* body mass index, *OR* odds ratio, *ASA* American Society of Anesthesiologists

## Discussion

This first international, multicenter, retrospective, cohort study comparing RDP and LDP in 542 patients with resectable pancreatic cancer from 33 centers in 11 countries found a comparable R0 resection margin and overall survival rate between RDP and LDP and a higher lymph node yield in RDP. Other notable differences were the lower conversion rate, higher rate of vascular resection, and higher rate of major complications in RDP, and a shorter operative time and shorter hospital stay in LDP. In multivariable analysis, RDP was not associated with major complications.

In recent years, MIDP has rapidly become the standard approach for symptomatic benign and low-grade malignant lesions requiring distal pancreatectomy.^1^ However, the oncological safety and efficacy of MIDP in patients with pancreatic cancer remains controversial and studies comparing RDP and LDP in patients with resectable pancreatic cancer are still scarce. First, the pan-European propensity score-matched DIPLOMA cohort study suggested that MIDP is associated with better short-term outcomes, i.e., less intraoperative blood loss and shorter hospital stay with a higher R0-resection rate, a higher lymph node yield, and comparable overall survival compared to ODP.^5^ Following on this, the same group recently completed the European, randomized, DIPLOMA-1 trial comparing MIDP and ODP in patients with resectable pancreatic cancer, and these results are expected soon.^23^ Recently, the first systematic review and meta-analysis comparing RDP with LDP in patients with pancreatic cancer included 6 retrospective studies, of which 5 single-center and 1 multicenter study, comprising a total of 572 patients (152 RDP, 420 LDP).^24^ The current study by itself included almost the same number of patients: 542 patients of 33 centers. The systematic review reported a higher R0 resection rate after RDP compared with LDP, without differences in operative time, tumor size, and lymph node yield. Only two studies, with in total 158 patients, reported on overall survival and found no differences between RPD and LDP.

The lower conversion rate in RDP as seen in the current study is in agreement with prior literature.^6,8,25–27^ This could be attributed to the technical capacity of the robotic platform, allowing for earlier and easier control of, for example, intraoperative bleeding, which may eventually be a reason for conversion. Furthermore, one-third of all conversions during LDP were emergency conversions against no emergency conversions during RDP. A previous study revealed that emergency conversions during MIDP are associated with increased overall morbidity and worse oncological outcome.^10^ A reduced conversion rate in RDP could be advantageous in this regard and therefore should be taken into consideration in the choice for the surgical approach of a distal pancreatectomy in patients with pancreatic cancer.

Remarkably, although the rate of major complications was 10% higher in the RDP group, the rates of postoperative pancreatic fistula, post-pancreatectomy hemorrhage, and delayed gastric emptying grade B/C were comparable between RDP and LDP. In multivariable, regression analysis, only an ASA III/IV classification was associated with major complications, a finding that has been described in previous literature.^28,29^ Also, there were proportionally more vascular resections performed in the RDP group (7.6% vs. 2.7%, *p* = 0.030). Although a correlation could not be proven, literature does suggest an association between vascular resections and major complications.^30^ On the other hand, the higher complication rate could be due to surgeons performing RDP during the first phase of their learning curve. Previous studies have proven that adoption of minimally invasive pancreatic surgery during the learning curve may cause increased morbidity rates.^31,32^ Unfortunately, this could not be verified in the present study, because no data were available on individual surgeons’ volume.

Regarding the oncological outcomes, a comparable R0-resection rate and higher lymph node yield was found after RDP compared with LDP. These results contradict the most recent systematic review, which reported a comparable lymph node yield and a higher R0 resection in RDP.^24^ However, the obtained difference should be interpreted with caution, given that it could possibly be influenced by differences in pathological examination protocols between centers rather than the quality of lymphadenectomy. For example, Sahakyan et al. demonstrated an increase in lymph node yield from 7 to 18 by standardizing the pathology examination without changing the surgical technique.^33^ In addition, the clinical relevance of the difference of only two lymph nodes could be questioned here, as no difference in positive lymph nodes or survival were observed between both groups. The comparable overall survival rates and disease-free intervals between RDP and LDP align with the results of a prior study that investigated the long-term outcomes between RDP and LDP in patients with pancreatic cancer in the National Cancer Database.^6^ These results indicate that the choice of approach does not impact patients’ survival.

The results of this study should be interpreted in light of several limitations. First, the retrospective design may have impacted the results as selection bias might be present and some important data were not available, such as on resection of Gerota’s fascia. Resection of Gerota’s fascia during distal pancreatectomy may improve oncological outcomes and therefore can have distorted the current comparison of both techniques.^34^ Second, no data on type of (neo)adjuvant treatment was available, although the use of neoadjuvant and adjuvant treatment in patients undergoing RPD and LDP was similar. FOLFIRINOX as adjuvant treatment has recently been associated with better overall survival in patients with resectable pancreatic ductal adenocarcinoma,^35^ so the obtained survival rates might be rather a reflection of this than the surgical technique. Third, the large number of centers participating in the study might have introduced heterogeneity. Although all participating centers had at least performed 50 MIDP procedures, their surgical technique as well as their experience on treating pancreatic cancer might differ. This also applies to the length of hospital stay, as outcomes may have varied due to different hospital discharge policies. Propensity score matching was considered for the current study, but eventually not performed due to comparable RDP and LDP groups and the potential loss of statistical power of matching. Fourth, no data on operative costs were collected. This is relevant given the high costs of the robotic system and also should be a topic in future prospective studies. A main strength of this study is the large sample size with a large number of centers reflecting current practice in 33 experienced centers from 11 countries.

## Conclusions

This international cohort study, which compared RDP with LDP in patients with resectable pancreatic cancer in experienced centers, is the largest, retrospective cohort to date. It suggests that RDP is as oncologically safe as LDP by showing comparable R0-resection and survival rates with a higher lymph node yield. Because prospective studies comparing RDP with LDP are still lacking, future randomized studies, which could have a noninferiority design, are needed to prevent selection bias and identify those patients who will benefit from the potential advantages of a robot-assisted procedure.
